# Advances in Antarctic Research for Antimicrobial Discovery: A Comprehensive Narrative Review of Bacteria from Antarctic Environments as Potential Sources of Novel Antibiotic Compounds Against Human Pathogens and Microorganisms of Industrial Importance

**DOI:** 10.3390/antibiotics7040090

**Published:** 2018-10-19

**Authors:** Kattia Núñez-Montero, Leticia Barrientos

**Affiliations:** 1 Laboratorio de Biología Molecular Aplicada, Centro de Excelencia en Medicina Traslacional, Universidad de La Frontera, Avenida Alemania 0458, 4810296 Temuco, Chile; k.nunez03@gmail.com; 2 Núcleo Científico y Tecnológico en Biorecursos (BIOREN), Universidad de La Frontera, Avenida Francisco Salazar 01145, 481123 Temuco, Chile; 3 Centro de Investigación en Biotecnología, Escuela de Biología, Instituto Tecnológico de Costa Rica, 30101 Cartago, Costa Rica

**Keywords:** Antarctic ecosystem, novel antibiotics, Antarctic bacteria, multidrug-resistant pathogens, extreme environment, bacterial cold-adaptation

## Abstract

The recent emergence of antibiotic-resistant bacteria has become a critical public health problem. It is also a concern for industries, since multidrug-resistant microorganisms affect the production of many agricultural and food products of economic importance. Therefore, discovering new antibiotics is crucial for controlling pathogens in both clinical and industrial spheres. Most antibiotics have resulted from bioprospecting in natural environments. Today, however, the chances of making novel discoveries of bioactive molecules from various well-known sources have dramatically diminished. Consequently, unexplored and unique environments have become more likely avenues for discovering novel antimicrobial metabolites from bacteria. Due to their extreme polar environment, Antarctic bacteria in particular have been reported as a potential source for new antimicrobial compounds. We conducted a narrative review of the literature about findings relating to the production of antimicrobial compounds by Antarctic bacteria, showing how bacterial adaptation to extreme Antarctic conditions confers the ability to produce these compounds. We highlighted the diversity of antibiotic-producing Antarctic microorganisms, including the phyla Proteobacteria, Actinobacteria, Cyanobacteria, Firmicutes, and Bacteroidetes, which has led to the identification of new antibiotic molecules and supports the belief that research on Antarctic bacterial strains has important potential for biotechnology applications, while providing a better understanding of polar ecosystems.

## 1. Introduction

The continual increase in global resistance to existing antibiotics, the prevalence of multidrug-resistant pathogens, and the rapid development of cross-resistance with new antibiotics have become a critical public health problem [1,2]. Bacterial antibiotic resistance can be acquired through mutation or horizontal transfer of a resistant gene, with phenotypic expression influenced by environmental and genetic factors [3]. All classes of antibiotics have experienced an emergence of resistance that compromises their use [4]. Hence, research efforts are aimed at discovering and producing novel and efficient therapeutic agents [5].

Regardless of efforts to produce new synthetic antibiotics [6], bioprospecting of natural environments has been responsible for the identification, production, and commercialization of most antibiotics [7] and it continues to provide key scaffolds for drug development [8]. Consequently, natural products—predominantly bacteria and fungi—are still the main sources for discovering novel antibiotics [7].

Recently, the chances of novel discoveries of bioactive molecules from various well-known bacteria have fallen dramatically. There are some hypotheses for this decrease in the discovery of new antibiotic molecules, including the large number of secondary metabolites that are cryptic or silent under common culture conditions, thereby inhibiting the identification of the total set of molecules from an isolate [9], and the frequent genetic exchanges between species that share similar chemical and physical selection factors in a particular environment [10,11], making the chances of obtaining new molecules from the same environment more difficult. As a result, isolating highly bioactive *Streptomyces* strains—a common source of multiple antibiotics—from different and untapped environments is recommended for identifying new antibiotic molecules. The exploration of new, unexplored habitats and uncommon environments has therefore become important for discovering novel bacterial antimicrobial metabolites [7,12,13].

Extreme environments are unusual, yet promising sources of novel antibacterial compounds. Bacteria living in adverse environmental conditions display atypical survival strategies, such as antagonistic activity that reduces the presence of competitive microorganisms, in order to achieve competitive advantages [14]. Such behavior is particularly necessary when nutrients are limited or difficult to uptake. Antarctic microorganisms and other extremophiles could have developed physiologically unique features, including chemically complex biosynthesized metabolites, to ensure their survival in complex habitats, as it has been also exemplified for halotolerant marine microorganisms [15]. Moreover, genomic sequencing reveals that microbes with large genomes—usually inhabiting complex harsh environments [16]—have the capacity to produce about ten times as many secondary metabolites as was previously recognized [8].

The antagonistic properties of organisms inhabiting extreme environments have not been investigated as extensively as they have for mesophiles [17]; however, the traditional approach of isolating and cultivating new microorganisms from underexplored habitats is still productive [6]. In recent years, research has exploited untapped and extreme environments, including the polar regions, complex marine habitats, and hyperthermal environments, to discover novel bioactive compounds with antibacterial, antifungal, and antitumor properties, and apply them to current clinical challenges [15,18,19,20,21]. This review summarizes the findings from research on Antarctic bacteria producing antimicrobial compounds, in order to: (i) Highlight the importance of the Antarctic ecosystem as a promising source for discovering novel antibiotics, (ii) demonstrate the significance of a growing body of research focused on antimicrobial activity of Antarctic bacteria, and (iii) highlight the strains already isolated from different Antarctic regions and their potential application against human pathogens and phytopathogens and in other industrial and pharmaceutical applications. Literature search was no systemic, conducted through Web of Science databases (Core Collection and All Databases) using search parameters as follows: TOPIC = (antibiotic * or antimicrobial *) and (Antarctic or polar or extreme environment *). Articles where antimicrobial activity was reported from Antarctic bacterial strains were selected and included in this review.

## 2. The Antarctic Environment Supports Varied Microbial Life

The Antarctic is separated from other continents by the Southern Ocean and the Antarctic Circumpolar Current [22]. It is a rocky land mass almost completely covered by an ice sheet, with regions of cold desert soils and freezing temperatures. Only about 2% of continental Antarctica is ice-free [23]. The massive ice sheet reflects 40–90% of the incidental solar radiation, causing a mass of cold dense air to accumulate on the polar plateau that sustains low polar temperatures. During the summer months, ice on the soils is subjected to thawing [24,25].

Consequently, Antarctica is known as the coldest, driest, windiest, and most inaccessible continent on the Earth [23]. In winter, temperatures at the South Pole reach a minimum of −80 °C to −90 °C in the interior, and a maximum of 5 °C to 15 °C near the coast in summer. The higher elevation of east Antarctica makes it colder than its western counterpart [23]. Only cold-adapted organisms survive in Antarctica, including penguins, seals, nematodes, tardigrades, mites, tundra vegetation, and many types of algae and other microorganisms [23].

Although the Antarctic region has been considered to be a natural, unexploited environment, persistent pollutants have been reported, including heavy metals, antibiotics, pesticides that could have been transported through natural processes by air and water currents, as well as improper waste disposal practices and/or incineration from research stations [26,27]. Furthermore, recent studies show that scientists and tourists have introduced non-indigenous microorganisms to the Antarctic, with implications for natural genetic diversity, mainly due to the transference of antibiotic resistance [28,29]. Interestingly, active volcanoes have been confirmed as a local source of heavy metals (cadmium, zinc, vanadium, arsenic, and gold) [30]; however, the atmospheric transport of pollutants, such as mercury, continues to be considered a major pathway for Antarctic environment contamination [31,32].

The survival and growth of organisms in this region is restricted not only by low temperatures, but also by the availability of water and associated osmotic stress, substratum instability, and frequently introduced contaminants [24]. In spite of these conditions, prokaryotes are the dominant biomass component in most Antarctic ecosystems and play a major role in the food web, biogeochemical cycles, and mineralization of pollutants [33]. Microorganisms have been detected in all habitats, including lakes, ponds, rivers, streams, rocks, and soils [33].

Proteobacteria (a major phylum of Gram negative bacteria) have been frequently found in Antarctic soils [34]. Nevertheless, their habitats differ with respect to multiple physical–chemical parameters, including nutrients, water activity, range of temperature, and consequently, microbial flora [33]. Studies that focused on microbial diversity in Antarctic soils have reported Proteobacteria and Actinobacteria as the most abundant phyla, while the phyla Cyanobacteria and Firmicutes were common but less frequent [35,36,37,38]. Because Firmicutes comprise several spore-forming genera, it is highly reasonable to believe that this bacterial group is predominant in the harsh Antarctic environment, since it can survive under severe conditions for long periods of time [39].

To our knowledge, there are no studies to date representing the total microbial composition of the Antarctic. Nonetheless, a recent multi -omics approach (culture-independent technique) showed the prokaryotic community occurring within the active layer at Edmonson Point, an ice-free area on the eastern slope at the foot of Mount Melbourne (Northern Victoria Land, Antarctica) [40]. The composition of this area—from more to less abundant—showed Proteobacteria, Actinobacteria, Acidobacteria, Bacteroidetes, Planctomycetes, Chloroflexi, and Verrucomicrobia [40], while Proteobacteria, Actinobacteria, Bacteroidetes, and Firmicutes have been more frequently reported through cultivation-based methods [17,34,41]. Interestingly, 16SrRNA sequencing and analysis revealed that anaerobic spore-forming Firmicutes have been shown to be the most abundant group in the Antarctic vascular plants rhizosphere [42].

Cultivable Antarctic bacteria have been frequently isolated using different media, depending on the group of interest, as was described for other environments, such as marine broth or agar for halotolerant [14], Luria–Bertani, tryptone soy [17,43], and R2A agar [34] for broad range culture. Diluted media, M1, oatmeal agar [44,45], among other culture media, have been recommended for the specific isolation of Actinobacteria. Even though Antarctic bacteria grow under low temperatures and survive freezing, optimal growth temperatures for isolates usually range from 4 °C to 20 °C, since Antarctic bacteria includes psychrotrophic and psychrophilic organisms [17]. Aerophyllic isolation conditions are usually preferred; therefore, aerobic organisms are typically isolated. However, microaerophilic bacterial strains, containing the anaerobic global regulator gene (*anr*), have been also reported [46], as well as obligate anaerobes [47,48].

Antarctic marine soils are free of snow and ice during the summer months and receive nutrients from the ocean and animal life—penguin guano, for example, enriches the soils with nitrogen and phosphorus, thus creating an environment capable of supporting abundant microbial growth [24]. O’Brien et al. [17] also show that all ornithogenic soil samples yielded high bacterial counts (6–9 log CFU·g^−1^) in comparison to other samples. Very high counts (10 log CFU·g^−1^) have been reported in ornithogenic soil through direct microscopic counting [49].

The presence of frozen water in Antarctic soils often leads to an increase in soil solute concentration, resulting in the creation of a hypersaline microenvironment [50]. Consequently, bacterial isolation in terms of colony-forming units from maritime Antarctica soils is higher in NaCl-containing media [17], showing the predominance of halotolerant bacteria in this habitat. In addition, high altitude soils in the Trans-Antarctic Mountains provide the least hospitable environment for microbial growth, since temperatures rise above freezing for only brief periods each year. Predictably, soils at over 1000 m above sea level contained low levels of microorganisms [17].

Although research on Antarctic microbial ecology began in the early 1900s [24], the region is still considered to be a potentially exploitable ecosystem for the discovery of new microorganisms with potential benefits for humans, due to its vast, unexplored diversity and peculiar environmental conditions. Bacteria with antimicrobial activity have been isolated from various Antarctic habitats, predominantly soils and seawater (Table 1). Higher antibiotic activity is typically produced by attached bacteria rather than their free-living counterparts in the pelagic zone [51,52]. This phenomenon might be due to the production of antimicrobial metabolites, which in a free-living stage could produce needless energy waste. Antagonistic interactions could play a key role in colonizing an ecological niche with high densities of bacteria [51,53], preventing other bacteria from colonizing particles. Although occurring less frequently, antimicrobial activity from free-living marine bacteria has also been reported and is reviewed in the following sections.

## 3. Bacterial Adaptation to Antarctic Extreme Conditions Might Confer the Ability to Produce Diverse and Novel Antimicrobial Compounds

Due to their extreme environment, Antarctic bacteria have been reported as a potential source for antimicrobial compounds [61]. Microorganisms need to develop different strategies to survive polar conditions [76,77], such as lack of substrate, high UV-radiation, sustained low temperatures, and short-term intense heat during the Antarctic summer [23]. These complex conditions represent diverse challenges for bacterial life, including reduced enzyme activity, decreased membrane fluidity, altered transport of nutrients and waste products, decreased rates of transcription, translation, and cell division, protein cold-denaturation, inappropriate protein folding, and intracellular ice formation [78].

Therefore, genetic adaptation of Antarctic microorganisms to polar stress factors would be expected to generate novel metabolic pathways through evolutionary selection [76]. Thus, one can suggest the synthesis of new metabolites from Antarctic bacteria with unique structures and specific biological activity, produced as a competitive advantage for niche colonization by establishing antagonistic relationships [23].

Such antagonistic interactions among Antarctic bacteria have been demonstrated [79], and numerous Antarctic bacterial isolates exhibited antagonistic properties against marine bacteria from the same environment [80]. Differences were observed in the inhibition patterns of single isolates, suggesting that their activity was more likely strain-specific rather than dependent on phylogenetic affiliation [79]. Similarly, five Actinobacteria strains were isolated from surfaces of Antarctic macroalgae and showed activity against other macroalgae-resident bacteria, both Gram-negative and Gram-positive strains [80]. This data suggest the potential exploitation of Antarctic bacteria as a novel source of antimicrobial compounds and confirms antagonistic interactions within bacterial strains in Antarctic marine ecosystems.

Reports on *Pseudoalteromonas* spp. also support the case for discovering Antarctic bacteria suitable for the production of antimicrobial metabolites. This genus has become a model for studying bacterial adaptation to cold, since it is frequently isolated from marine polar regions [81,82,83]. Genomic analysis of the Antarctic strain *Pseudoalteromonas haloplanktis* TAC125 has demonstrated important features relating to its adaptability [84]. Among them a specific codon usage bias, which is involved in the resistance to protein aging features including asparagine cyclization and deamination [85]; the resistance to reactive oxygen species (ROS), which represent a significant stressor under cold conditions [86,87]; and the elevated number of rRNA and tRNA genes, which could explain its translational efficiency even in cold conditions [67]. Additionally, cryotolerance and osmotic stress resistance have been conferred by the enhanced uptake of compounds including: Spermine, Glutathione, Ornithine, and others, related to Glutathione metabolism; increased metabolic potential in terms of Arginine and Proline metabolism, β-Alanine metabolism, fatty acid uptake, and biosynthesis; and protein S-thiolation [88]. Similarly, carotenoid pigmentation confers protection to Antarctic bacteria against freeze-thaw cycles and solar radiation [89].

Another approach using the existing genomes of *Pseudoalteromonas* isolates has shown important evolutionary implications regarding the diverse genetic information of cold-adapted bacteria, including the relatively high number of genes obtained through horizontal gene transfer—12,981 genes (14% of the genome) were associated with mobile genetic elements—and an extensive evolutionary genome reduction of this genus [84]. These modifications probably acted as the main factors leading to the genomic diversity between *Pseudoalteromonas* strains, and might correspond to other isolated Antarctic bacteria.

On the other hand, it is reasonable to believe that strains isolated from the Antarctic might possess genes involved in the adaptation to cold temperatures, such as Anti-Freeze Proteins (AFPs), a class of proteins able to inhibit ice nucleation or decrease the water freezing point temperature [90,91]. Remarkably, recent studies revealed no clear distinction between Antarctic and non-Antarctic *Pseudoalteromonas* strains, with all of them showing four to five AFPs [84]. This could be a genus-specific feature.

Regarding antimicrobial compounds, Bosi et al. [84] demonstrated that Antarctic *Pseudoalteromonas* genomes harbor at least one cluster involved in bioactive molecule synthesis, with the number of clusters per genome ranging from 1 to 19. As expected, high variability of biosynthetic operons was confirmed in terms of number and composition across the different genomes [84]. Furthermore, experimental evidence has shown the production of broad-spectrum antimicrobial compounds by Antarctic Actinobacteria preferentially at lower temperatures [60]. This behavior and genetic evidence supports the assumptions that Antarctic bacteria use antimicrobial compounds as an advantage to compete for nutrients and space in the cold environment.

## 4. Antarctic Exhibits Significant Diversity of Antibiotic-Producing Bacteria with Pharmaceutical Applications

### 4.1. Proteobacteria Phylum Members

A screening for bioactive secondary metabolites showed that 25% (14 out of 57) of Proteobacterium species isolated from Antarctic soils generate an extracellular crude extract that is highly bioactive against *Candida albicans* and *Staphylococcus aureus*. The most bioactive genus was *Bradyrhizobium*, which contributed 64% of the total bioactive isolates in the study (9 out of 14) [11]. However, genera *Methylobacterium*, *Paracoccus*, and *Sphingomonas* were bioactive as well [11]. Other screenings also report the antimicrobial activity of *Halomonas* sp. strains against a broad range of human pathogens, including the Gram negative *Salmonella enterica*, *Escherichia coli*, *Enterobacter aerogenes*, *Citrobacter freundii*, *Serratia marcescens*, *Shigella flexnerii*, *Shigella sonnei*, *Staphylococcus aureus*, and *Staphylococcus epidermidis*, and the Gram positive *Enterococcus faecalis* and *Listeria monocytogenes* [63].

Asencio et al. [61] reported on a *Janthinobacterium* sp. ethanolic extract presenting antibacterial activity against multidrug-resistant bacteria isolates from Chilean hospitals. Among them were *Acinetobacter baumannii* and *Pseudomonas aeruginosa*, both carbapenemase producers; *E. coli* and *Klebsiella pneumoniae*, producing extended-spectrum β-lactamases; and two multiresistant strains of *Serratia marcescens*, producing a chromosomal AmpC beta-lactamase (the MIC values ranged between 0.5 and 16 μg·mL^−1^) [61]. *Janthinobacterium* sp. extract activity against *Mycobacterium smegmatis*, *Mycobacterium tuberculosis* [62], *Bacillus subtilis*, *Bacillus cereus*, *Candida* sp., and *Cryptococcus* sp. [56] have been reported as well.

Similarly, members of *Pseudomonas* genus were found to produce proteinaceous compounds active against Gram positive bacteria (*Listeria* spp., *B. subtilis*, *Bacillus cereus*, *Sarcina lutea*, *Micrococcus luteus*, and *Brochothrix thermosphacta)*, Gram negative bacteria (*P. aeruginosa*, *E. coli*, *Acinetobacter johnsonii*, *S. enterica*, *K. pneumoniae*, *Enterobacter cloacae*, and *Vibrio parahaemolyticus*) and yeast (*Candida* sp., *Cryptococcus* sp.) [17,56,69]. Another *Pseudomonas* sp. strain isolated from marine sediments showed activity against *Staphylococcus aureus* [38]. Additionally, volatile and soluble compounds against numerous members of *Burkholderia cepacia* complex (Bcc) pathogens have been reported for other *Pseudomonas* sp. isolates [54]. This activity was also described for *Psychrobacter* sp. strains obtained from marine sediments [54]. Interestingly, a *Burkholderia* sp. strain isolated from ornithogenic soil showed antimicrobial potential against some Gram positive (*B. subtilis* and *B. cereus*) and Gram negative (*A. johnsonii*) bacteria [56].

Strains belonging to *Pseudoalteromonas* genus expressed antagonistic activity against *E. coli* and *Proteus mirabilis* [14] and several members of Bcc [65], inhibiting their growth primarily due to the synthesis of numerous volatile compounds (VOCs). Remarkably, this study reported a constitutive synthesis of the volatile antimicrobial compounds (not induced by the presence of target strains), suggesting that production of such molecules is a common feature for the Antarctic strains [65]. Additionally, *P. haloplanktis* TAC125 produces a saccharidic compound that lacks antibacterial activity against free-living bacteria, but exhibits specific activity against biofilms formation of *S. epidermidis* strains isolated from infected catheter and septic arthritis [66]. Other studies have identified antibiotic compounds from different *Pseudoalteromonas* sp. strains isolated from marine environments, including extracellular active agents [92], molecules against methicillin-resistant pathogens [93], and genetic clusters related to the synthesis and regulation of antimicrobial compounds [94].

Since different *Pseudoalteromonas* sp. strains have shown important bioactive characteristics, this taxon is used as a model for molecular studies on the potential of Antarctic bacteria. In particular, Antarctic isolates have a great number of biosynthetic operons producing antimicrobial compounds, and pigmented *Pseudoalteromonas* sp. strains are characterized by the presence of a higher number of operons [84]. This result agrees with bioactivity assays where pigmented *Pseudoalteromonas* sp. exhibited the largest inhibition [95]. Other authors have highlighted the antimicrobial potential of pigmented bacteria from Antarctic marine environments [80]. Consequently, it has been proposed that those strains are able to produce a wider array of bioactive molecules, since they have a larger number of antimicrobial-producing operons [84].

Additionally, novel Proteobacteria species with antimicrobial activity have been found in the Antarctic. *Lysobacter oligotrophicus* was first described as a new member of the genus *Lysobacter*, which are strongly proteolytic and characteristically lyse a variety of microorganisms, as well as nematodes. *L. oligotrophicus* produced esterase, amylase, and protease, and showed a capacity to lyse Gram negative bacteria (*E. coli*, *Lysobacter enzymogenes*, and *Rhodoligotrophos appendicifer*) and *Saccharomyces cerevisiae*. Indeed, autolysis of this strain was observed in the stationary phase [64].

### 4.2. Actinobacteria Phylum Members

It is well known that bacteria from the phylum Actinobacteria represent a prominent source of biologically active natural compounds, since they produce versatile secondary metabolites [8]. Moreover, Actinobacteria are one of the major phyla of the domain bacteria, comprising 17 orders [96,97]. Remarkably, Actinobacteria comprise the genus *Mycobacterium* (a deadly bacterial pathogen), but also *Streptomyces* genus, which are most important for antibiotic production and are responsible for two-thirds of all known antibiotics [98].

Katz and Baltz [8] reported that bacteria belonging to this phylum have the capacity to produce around 30 to 50 secondary metabolites, making them one of the major sources of natural compounds. However, in recent years, the discovery of new antibiotic molecules from Actinobacteria has been infrequent, in part due to the recurrent genetic exchange between species from similar ecosystems [10]. As a result, the latest studies are focused on a search for Actinobacteria from unusual and isolated environments in order to discover novel antimicrobial metabolites [99,100].

Antarctic investigations have shown promising results with antimicrobial active compounds from Actinobacteria. A *Streptomyces* sp. strain capable of inhibiting the growth of seven Gram negative food borne pathogens (from genera *Vibrio*, *Enterobacter*, *Klebsiella* and *Salmonella*) and eight Gram positive food borne pathogens (from genera *Listeria*, *Bacillus*, *Enterococcus* and *Staphylococcus*) was isolated from Antarctic soil [60]. Likewise, strains of *Arthrobacter* sp. were found to produce active compounds against a broad range of human pathogens, including Gram positive, Gram negative, yeast (*B. subtilis*, *B. cereus*, *M. luteus*, *P. aeruginosa*, *A. johnsonii*, *Candida* sp., *Cryptococcus* sp., *Listeria* spp. and *B. thermosphacta*) [17,56] and several Bcc strains [54]. Moreover, 22 marine Antarctic strains belonging predominantly to *Arthrobacter* sp., *Janibacter thuringensis*, *Nesterenkonia* sp., and *Rhodococcus fascians* expressed antagonistic activity against *E. coli*, *M. luteus*, *B. subtilis*, and *P. mirabilis* [14]. Similar results have been reported for other *Rhodococcus* sp. strains isolated from Antarctic ornithogenic soils [56].

Lee et al. (2012) [57] reported extracellular bioactive metabolites from 38% (15 out of 39) of Actinobacterial isolates, inhibiting the growth of *C. albicans*, methicillin-resistant *S. aureus*, and *P. aeruginosa*. *Brevibacterium* genus showed the highest activity, with five bioactive isolates [57]. Although the 15 isolates with antibiotic activity were obtained from soil samples at the same location, the isolates turned out to be taxonomically diverse, since the strains belong to 11 different genera (*Nocardioides*, *Gordonia*, *Micromonospora*, *Demetria*, *Rhodococcus*, *Janibacter*, *Dermacoccus*, *Kocuria*, *Lapillicoccus*, and *Brevibacterium*). These findings confirm the abundant bacterial diversity in Antarctic environments. Comparably, *Gordonia terrae*, *Leifsonia soli*, and *Terrabacter lapilli* isolated from Antarctic volcanic soils showed inhibitory activity against *S. enterica* serotype Paratyphi and *S. enterica* serotype Enteritidis [58]. Other marine Actinobacteria have been reported to have antimicrobial activity against numerous human pathogens. In fact, novel species have been found to exhibit at least one of the biosynthetic genes coding for polyketide synthases and non-ribosomal peptide synthetases [18] related to the production of these secondary metabolites [101].

Furthermore, *Nocardioides* sp. belonging to Actinobacteria phylum isolated from Antarctic soil showed extracellular antimicrobial activity against Gram positive and Gram negative bacteria, especially *S. aureus* and *B. subtilis*. Preliminary analysis showed that the compounds with antimicrobial activity produced by this strain are mainly glycolipids and/or lipopeptides. Remarkably, the type of compound and specific activity against *S. aureus* depended on the used carbon source [59]. This finding is important because the antimicrobial substances might be produced mainly by the secondary metabolite pathway, depending on nutritional conditions and probably based on different chemical signals from the environment, as reported for other Antarctic isolates [55,68,102]. Hence, nutritional factors, such as carbon and nitrogen sources, metal ions, and inorganic phosphate in the media, may affect the production of secondary metabolites during cultivation of the microorganisms. This could be a critical aspect to consider for screening and discovering antimicrobial compounds produced by bacteria.

### 4.3. Other Phylum Members

A dominance of Cyanobacterial diversity in Antarctic and other polar environments has been reported by Vincent [103] and Zakhia et al. [104], indicating that the search for antimicrobial compounds from Antarctic Cyanobacteria seems to have a promising future. Interestingly, novel Antarctic Cyanobacteria was described as having antibacterial/antifungal potential. Some unknown and uncultured Cyanobacterial strains with antibiotic activity were found through 16S ribosomal RNA gene (16S rRNA) and ITS sequence analysis [104]. From 48 total isolates, 35% of the strains (identified as *Pseudophormidium* sp., *Phormidium priestleyi*, *Leptolyngbya antarctica*, *Nostoc* sp. and *Phormidium murrayi*) showed activity against *S. aureus*, or the fungi *Aspergillus fumigatus* and *Cryptococcus neoformans* in a strain-specific manner, using ethyl acetate and methanol crude extracts [71,105]. Activity against *M. tuberculosis*, *S. aureus*, *S. typhi*, *P. aeruginosa*, *Enterobacter aerogenes* and multidrug-resistant strains of *E. coli*, was also reported for extracts of Antarctic *Nostoc* sp. strains [72].

On the other hand, bioactivity of Firmicutes phylum has been occasionally reported from Antarctic isolates; however, O’Brien et al. [17] reported for the first time the isolation of *Planococcus* sp. strains from Antarctic soil with the production of bioactive compounds against Gram positive human pathogens (*Listeria* spp. and *B. thermosphacta*). In addition, a proteinaceous compound from *Enterococcus* sp. isolated from penguin rookeries and belonging to Firmicutes phylum exhibited a strong effect against multidrug-resistant strains of *C. albicans* [75]. *Bacillus* sp. and *Sporosarcina* sp. strains isolated from Antarctic soils demonstrated activity against yeast (*Candida* sp. and *Cryptococcus* sp.), Gram positive (*B. subtilis*, *B. cereus*, *S. lutea*, and methicillin resistant *S. aureus*) and Gram negative bacteria (*P. aeruginosa*, *E. coli*, and *A. johnsonii*) [56,74].

Similarly, Mojib et al. [62] first described an Antarctic *Flavobacterium* sp. belonging to Bacteroidetes phylum, with activity against *M. smegmatis* and *M. tuberculosis*. In addition, an Antarctic *Pedobacter* sp. strain, a member of this phylum, was reported for the first time as producing inhibitors against human pathogens (*E. coli*, *S. enterica*, *K. pneumoniae*, *E. cloacae*, and *B. cereus*) [69]. Additionally, in a high throughput screening for antimicrobial activity, 675 strains were isolated from 17 samples of benthic mats collected from 10 lakes located in three distinct Antarctic regions and 18% were active against at least one of the panel pathogens (*S. aureus*, *E. faecium*, *and E. coli*). Some of the active bacteria were members of phyla Actinobacteria and Proteobacteria; however, most of the isolates belong to Bacteroidetes, while others could not be assigned to any classification based on 16S rRNA analysis [55].

## 5. Antarctic Bacterial Strains Exhibit Activity against Microorganisms of Industrial Importance

Unlike the inhibitors produced by mesophylls, the antimicrobials produced in cold environments, such as the Antarctic, need to function at low temperatures for the organisms to gain a competitive advantage during their growth cycle. Such cold-active antimicrobial compounds may be exploitable in industrial applications, including chilled-food preservation [17].

Furthermore, Antarctic bacterial isolates have shown potential against biofilm formation, which is of great importance for different industrial applications. For example, Leyton et al. [70] reported that 26.9% of Antarctic bacterial isolates (from a total of 67) strongly inhibited the biofilm of *Flavobacterium psychrophilum*, with the greatest effect shown by proteins (48–56 kDa) secreted by *Pseudomonas fragi* strains. *F. psychrophilum* affects farmed salmon and trout worldwide and its ability to form biofilms, in addition to the emergence of resistant strains, have increased the resistance to antibiotics [70].

Additionally, Antarctic antibiotic producers could inhibit other nonhuman pathogens, including plant pathogens. A *Bacillus* sp. strain, belonging to the phylum Firmicutes, was isolated from Antarctic seawater and showed strong antifungal activity against several plant pathogenic fungi, including *Paecilomyces variotii*, *Colletotrichum gloeosporioides*, *Fusarium oxysporum*, *Trichoderma viride*, *Rhizoctonia solani Kühn*, *Alternaria longipes*, and *Sclerotinia sclerotiorum* [73]. Some *Bacillus* species, other than Antarctic and marine, have been reported to produce strong active antimicrobial compounds against phytopathogens [106,107,108].

Furthermore, *Nocardioides* sp. belonging to Actinobacteria phylum isolated from Antarctic soil showed extracellular antimicrobial activity against *Xanthomonas oryzae*, which is one of the most harmful diseases of rice, causing bacterial blight [59]. Similar results against these phytopathogens have been reported for other Actinobacteria isolates (*Arthrobacter* sp.), Proteobacteria (*Pseudomonas* sp. and *Burkholderia* sp.), and Firmicutes strains (*Sporosarcina* sp.) [56]. Additionally, at least three *Halomonas* sp. strains isolated from Antarctic sea water showed activity against multiple phytophatogen species, including *Xanthomonas axonopodis*, *Xanthomonas albilineans*, *Erwinia stewartii*, and *Erwinia amylovorans* [63]. This indicates that Antarctic strains represent a valuable and almost unexplored source of bioactive molecules for biocontrol of plant diseases, representing a worldwide need.

## 6. Recent Advances Identified New Antibiotic Molecules from Antarctic Bacteria

**Figure 1:**
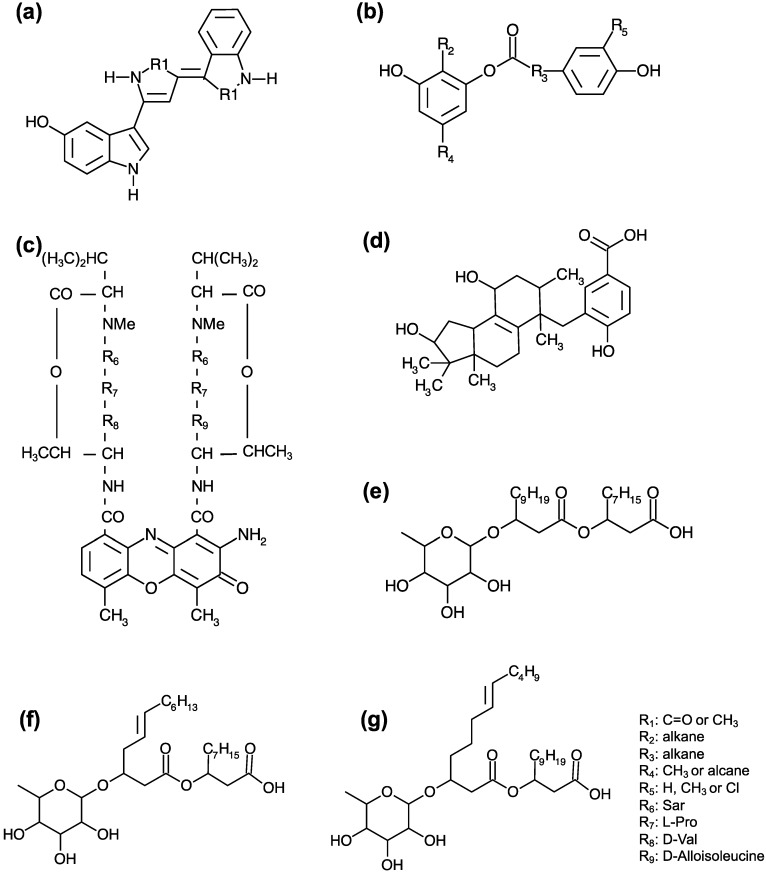
Chemical structures of molecules with antibiotic activity obtained from Antarctic bacteria: (**a**) *Streptomyces flavovirens*, (**b**) and (**c**) *Flavobacterium* sp., (**d**) *Nostoc* sp., and (**e**–**g**) *Pseudomonas* sp. All molecules, except (**e**), correspond to novel antimicrobial metabolites. Modified from: [23,54,62,72].

Although just a limited number of antibiotic molecules from Antarctica bacteria have been purified and described, most of them correspond to new molecules (Figure 1). In a study reporting an analysis of a total set of six antimicrobial-producing bacteria by high-resolution mass spectrometry, the resulting exact mass of the purified extracts did not match any known compound in the natural products library for any of the analyzed strains [55]. In addition, some of the purified fractions could not be associated with complete antimicrobial activity, suggesting that one Antarctic isolate may represent a source of numerous new antimicrobial compounds, which might act synergistically against competing microorganisms. Furthermore, the production of methylamine by *P. haloplanktis* TAC125, with anti-Bcc activity, was demonstrated by VOCs capture, accumulation, and storage, followed by solid-phase microextraction gas chromatography–mass spectrometry (SPME-GC–MS). Despite this finding, methylamine only partially contributes to the effect against Bcc; thus, the efficiency of marine Antarctic bacteria to inhibit almost all Bcc strains may be related to the synergistic effect of multiple unknown VOCs [68].

Additionally, an antibacterial molecule from the Antarctic Cyanobacterium *Nostoc* sp. was elucidated based on UV, IR, NMR, electron impact mass spectra (EIMS), and electronspray ionization mass spectra (ESIMS) data [72]. The structure of the active principle was proposed as 4-[(5-carboxy-2-hydroxy)-benzyl]-1,10-dihydroxy-3,4,7,11,11 pentamethyloctahydrocyclopenta<a>naphthalene, which is an intracellular biomolecule with broad-range antibacterial activity. This molecule is similar to anthraquinone and indane derivatives of a diterpenoid and different from the various biomolecules reported from *Nostoc* species other than of Antarctic origin. The rate of production of the active principle corresponds to 1.70 mg·g^−1^ biomass dry weight [72].

Another elucidated antibacterial compound structure corresponded to a chromophore 3-amino-1,8-dimethyl-2-phenoxazone-4,5-dicarboxylic acid connected with two cyclopentapeptides, which belongs to actinomycins and was produced by *Streptomyces flavovirens*, isolated from Antarctic soil samples, with antimicrobial and antitumor activity [23]. Additionally, Asencio et al. [61] reported the presence of violacein in an antibiotic ethanolic extract from *Janthinobacterium* sp., which also contains other undetermined compounds that can act as synergistic agents with violacein. The pigmented compound of the other Antarctic *Janthinobacterium* sp. strain was determined by NMR as (3-[5-(3-hydroxyl-1H-indol-3yl)-2-R1-1H-pyrrol-3-ylidene]-2-R1-1H-indol), with some differences for other violacein pigments previously reported [62]. In the same study, the structure of additional pigment obtained from Antarctic *Flavobacterium* sp. was elucidated and described as the first reported flexirubin with antimicobacterial activity [62].

Three other soluble compounds from *Pseudomonas* sp. strain with activity against Bcc strains and *S. aureus* were elucidated by high-resolution electrospray ionization mass spectrometry (HRESIMS), NMR, and LC-MS as rhamnolipids as follow: (1) C_28_H_52_O_9_, containing two fully saturated lipid chains; (2) C_28_H_50_O_9_, with one ester, one carboxylic acid group, two olefinic carbons, an anomeric carbon, A and B chains having 10 and 12 carbons, respectively, and a single unsaturation at B5; and (3) C_30_H_54_O_9_, with A and B chains and a single unsaturation at B7 [54]. The first compound was already known [109], but the last two compounds were reported as new rhamnolipids with antimicrobial activity. Rhamnolipids are well-known secondary metabolites synthesized by members of different Gram negative species. They perform several functions in bacteria, including surface motility and biofilm development [110]. Recently, they have emerged as potential antimicrobials against a broad range of pathogens, due to their intercalation into biological membranes leading to cell death by a permeabilizing effect [111].

## 7. Concluding Remarks

Bacteria isolated from the Antarctic environment are an unexploited source of novel antimicrobial compounds, which may be advantageous in food, therapeutic, health, and industrial applications in the future. Data about Antarctic microorganisms producing antimicrobial compounds are scant in comparison to other extensively studied environments. Although we exposed numerous bacterial strains from different phyla with various antibiotic activity, it is difficult to evaluate and compare the data generated from the different authors, due to the diversity of variable parameters in the screening process, such as isolation circumstances, target selection, and assay conditions. Consequently, it is important to increase investigations focused on antimicrobial activity from Antarctic bacteria in order to amplify comparable available data.

Despite most reports showing Antarctic bacterial strains as a source of antibiotic compounds, the samples/isolates lacked large-scale, high-throughput bioprospection, showing results only for a limited panel of target pathogens and usually just one condition—crude extract. Therefore, developing and applying robust screening models and high-throughput methods is of great importance for studying antimicrobial activity from single or multiple Antarctic bacterial strains in order to increase the chances for the discovery and identification of novel antibiotic molecules. To achieve this goal, it is imperative that the experimental design include all possible variables, such as recovering intra- and extracellular extracts produced in different growth conditions, using possible inducers of antimicrobial activity, and testing against a larger number of targets. To our knowledge, just one study exists where a robust screening model was applied, focusing on the search of antimicrobial activity from Antarctic bacteria, which analyzed 6348 extract samples that were tested in two or three media with different incubation periods and extraction methods [55].

**Figure 2:**
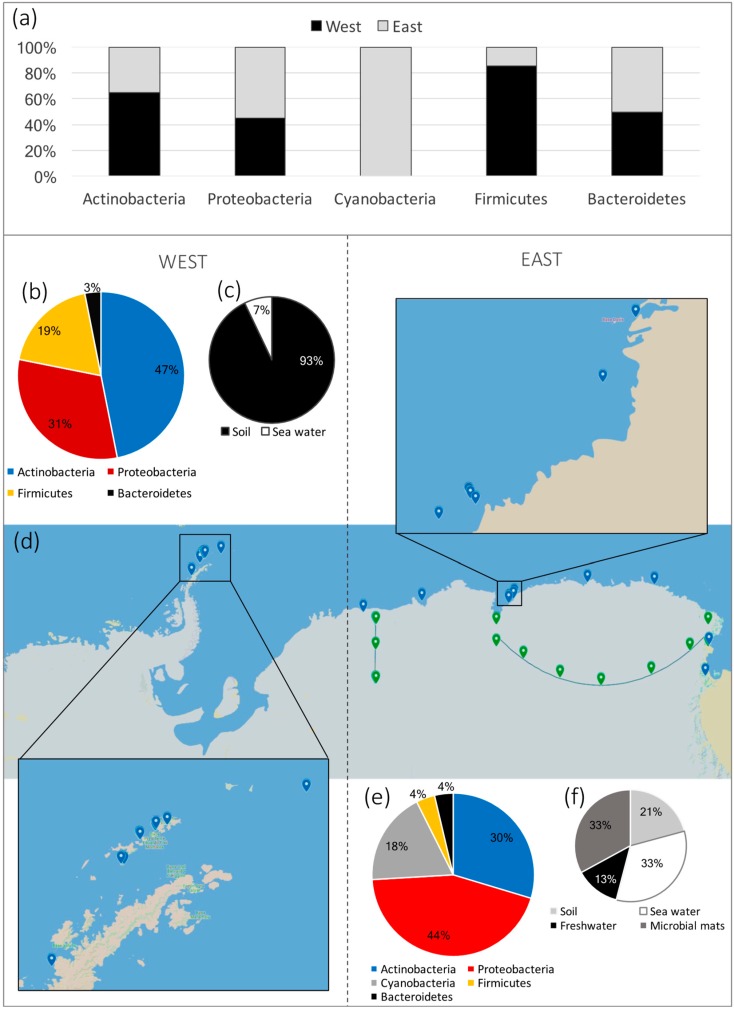
Reported diversity of Antarctic bacteria producing antimicrobial compounds and the geographical locations where diverse sample types were collected for isolation. Different phyla diversity has been reported for west and east Antarctic (**a**); each phylum has been reported with different frequency for the west side (**b**) compared to the east counterpart (**e**). Geographical locations where bacteria with antimicrobial activity have been isolated at each Antarctic side are shown, indicating that the west side has been less explored (**d**). Blue marks represent regions where punctual sampling was executed; green marks joined by a line illustrate two studies [14,17] where the complete area was sampled; the amplified squares show regions with multiple sampling sites. Active strains have been isolated utilizing different sample sources for west (**c**) and east (**f**) continent sides, including soil and seawater for both areas, and freshwater and microbial mats on the west side.

The search for Antarctic microorganisms that could have industrial applications started only in the late 20th century and relatively few scientific groups are pursuing this research. Since then, only a few antibiotic molecules have been purified and elucidated; however, most of them correspond to new molecules. As a result, further work is needed for the identification and purification of new strains, and characterization of the chemical structures of new antibiotics with pharmacological or industrial applications. Geographical locations already covered by research in the Antarctic are limited (Figure 2), particularly on the west side of the continent, and a clear-cut correlation is lacking between the antimicrobial activity pattern and taxonomical distribution of bacterial strains in the Antarctic regions [55]. Thus, there is a great opportunity to continue the discovery and characterization of new antimicrobials from unexplored Antarctic regions. Furthermore, this review does not consider reports of bacteria with antimicrobial activity isolated from Antarctic organisms. Nevertheless, there are reports of their ability to inhibit pathogens, for example for bacteria isolated from sponges [112,113,114] and macroalgae [115]. Therefore, there could be other unusual habitats within the Antarctic that are suitable for the discovery of antimicrobial compounds.

Finally, we have demonstrated the importance of better research on the inhibitory power of Antarctic strains from a clinical and industrial point of view, but additional Antarctic bioprospecting is necessary in order to understand the role of novel antibacterial molecules in structuring microbial, polar communities from an ecological point of view. There is little information available on the interactions between organisms in polar ecosystems, and very little is known in particular about the microbial diversity and functional capacity of the Antarctic permafrost compared to the Arctic, where the majority of comprehensive metagenomic studies have been done [116]. The application of -omics methods on Antarctic systems will provide an important understanding of microbial diversity, functional capacity of the ecosystem, novel antibiotic molecules, and their role in microbial community modulation. Moreover, as recently highlighted by Mocali et al. [88], multi-omics analyses are needed to generate information that could contribute to filling the gap between genotypic and phenotypic features relating to bacterial cold-adaptation mechanisms. In conclusion, research on Antarctic bacterial strains represents an important potential for biotechnology, while providing a better understanding of polar ecosystems.

## Figures and Tables

**Table 1:** Antimicrobial substance-producing bacteria from Antarctic regions.

Isolate Identification	Type of Sample	Place of Sampling	Antagonist Activity	Reference
**Actinobacteria**				
*Arthrobacter* sp.	Sea water	72°19′ S to 74°53′ S–163°48′ E to 70°16′ E: Stations Mergellina Santa Maria Novella, Tiburtina, Road Bay, Gerlache Inlet, Evans Cove, Inexpressible Island, Cape Hallet, and Tethys Bay	*Escherichia coli*, *Micrococcus luteus*, *Bacillus subtilis*, *Proteus mirabilis*	[14]
	Soil	72°19′ S to 77°83′ S–16° 55′ E to 17° 16′ E: Cape Hallett, Edmonson Point, Kay Island, Cape Russell, Lake Hoare, Harrow Peaks, Crater Circe, Battleship Promontory, Mount, McGee, Mount Rittmann, Mount Melbourne	*Listeria* spp., *Brochothrix thermosphacta*	[17]
	Marine sediment	74°41′36.96″ S, 164°6′42.12″ E: Terranova Bay, Ross Sea.	*Burkholderia cepacia* complex pathogens	[54]
	Benthic microbial mat	Larsemann Hills, Vestfold Hills and McMurdo Dry Valleys	*Staphylococcus aureus*, *Enterococcus faecium*, *E. coli*	[55]
	Ornithogenic soil	62°59′ S, 60°34′ W: Whalers Bay on Deception Island, South Shetland Islands and 65°14′44.6″ S, 64°15′26″ W: Galindez Island, Argentine Islands	*B. subtilis*, *Bacillus cereus*, *M. luteus*, *Pseudomonas aeruginosa*, *Acinetobacter johnsonii*, *Xanthomonas oryzae*, *Candida* sp., *Cryptococcus* sp.	[56]
*Brevibacterium* sp.	Soil	62°24′ S, 59°47′ W: Barrientos Island	*S. aureus*, *Candida albicans*	[57]
*Demetria* sp.	Soil	62°24′ S, 59°47′ W: Barrientos Island	*S. aureus*	[57]
*Gordonia* sp.	Soil	62°24′ S, 59°47′ W: Barrientos Island	*S. aureus*, *C. albicans*	[57]
*Gordonia terrae*	Soil	62°58′42.2″ S, 60°42′71.5″ W: Deception Island	*Salmonella enterica* serotype *Paratyphi*, *S. enterica* serotype *Enteritidis*	[58]
*Janibacter* sp.	Soil	62°24′ S, 59°47′ W: Barrientos Island	*S. aureus*	[57]
*Janibacter thuringensis*	Sea water	72°19′ S to 74°53′ S–163°48′ E to 70°16′ E: Stations Mergellina Santa Maria Novella, Tiburtina, Road Bay, Gerlache Inlet, Evans Cove, Inexpressible Island, Cape Hallet, and Tethys Bay	*E. coli*, *M. luteus*, *P. mirabilis*	[14]
*Kocuria* sp.	Soil	62°24′ S, 59°47′ W: Barrientos Island	*S. aureus*	[57]
*Lapillicoccus* sp.	Soil	62°24′ S, 59°47′ W: Barrientos Island	*C. albicans*	[57]
*Leifsonia soli*	Soil	62°58′42.2″ S, 60°42′71.5″ W: Deception Island	*S. enterica* serotype *Paratyphi*, *S. enterica* serotype *Enteritidis*	[58]
*Micromonospora* sp.	Soil	62°24′ S, 59°47′ W: Barrientos Island	*S. aureus*, *P. aeruginosa*	[57]
*Nesterenkonia* sp.	Sea water	72°19′ S to 74°53′ S–163°48′ E to 70°16′ E: Stations Mergellina Santa Maria Novella, Tiburtina, Road Bay, Gerlache Inlet, Evans Cove, Inexpressible Island, Cape Hallet, and Tethys Bay	*E. coli*	[14]
*Nocardioides* sp.	Soil	Casey Station, Wilkes Land	*S. aureus*, *B. subtilis*, *X. oryzae*	[59]
		62°24′ S, 59°47′ W: Barrientos Island	*C. albicans*	[57]
*Rhodococcus* sp.	Soil	62°24′ S, 59°47′ W: Barrientos Island	*S. aureus*	[57]
	Ornithogenic soil	62°59′ S, 60°34′ W: Whalers Bay on Deception Island, South Shetland Islands and 65°14′44.6″ S, 64°15′26″ W: Galindez Island, Argentine Islands	*B. subtilis*, *B. cereus*, *P. aeruginosa*, *Candida* sp., *Cryptococcus* sp.	[56]
*Rhodococcus fascians*	Sea water	72°19′ S to 74°53′ S–163°48′ E to 70°16′ E: Stations Mergellina Santa Maria Novella, Tiburtina, Road Bay, Gerlache Inlet, Evans Cove, Inexpressible Island, Cape Hallet, and Tethys Bay	*E. coli*, *M. luteus*, *B. subtilis*, *P. mirabilis*	[14]
*Streptomyces* sp.	Soil	62°12′26.4″ S, 58°58′28.7″ W: Fildes Peninsula, King George Island.	*Vibrio parahaemolyticus*, *S. enterica* serovar *Typhimurium*, *Enterobacter cloacae*, *Klebsiella pneumoniae*, *B. cereus*, *Listeria monocytogenes* (serotypes I, IV and aa), *Enterococcus. faecalis*, *Staphylococcus haemolyticus*, *Staphylococcus equorum*, *S. aureus*	[60]
*Terrabacter lapilli*	Soil	62°58′42.2″ S, 60°42′71.5″ W: Deception Island	*S. enterica* serotype *Paratyphi*, *S. enterica* serotype *Enteritidis*	[58]
**Proteobacteria**				
*Bradyrhizobium* sp.	Soil	62°24′ S, 59°47′ W: Barrientos Island	*C. albicans*	[57]
*Burkholderia* sp.	Ornithogenic soil	62°59′ S, 60°34′ W: Whalers Bay on Deception Island, South Shetland Islands and 65°14′44.6″ S, 64°15′26″ W: Galindez Island, Argentine Islands	*B. subtilis*, *B. cereus*, *A. johnsonii*, *X. oryzae.*	[56]
*Janthinobacterium* sp.	Soil	62°12′ S, 58°57′ W: Fildes Peninsula, King George Island	*Acinetobacter baumannii*, *P. aeruginosa*, *E. coli*, *K. pneumoniae*, *Serratia marcescens*	[61]
	Freshwater	70°45′52.3″ S, 11°37′10.7″ E: Lake Podprudnoye, Schirmacher Oasis, Dronning Maud Land	*Mycobacterium smegmatis*, *Mycobacterium tuberculosis*	[62]
	Benthic microbial mat	Larsemann Hills, Vestfold Hills and McMurdo Dry Valleys	*S. aureus*, *E. faecium*, *E. coli*	[55]
	Ornithogenic soil	62°59′ S, 60°34′ W: Whalers Bay on Deception Island, South Shetland Islands and 65°14′44.6″ S, 64°15′26″ W: Galindez Island, Argentine Islands	*B. subtilis*, *B. cereus*, *P. aeruginosa*, *E. coli*, *Candida* sp., *Cryptococcus* sp.	[56]
*Halomonas* sp.	Sea water	57°59′422” to 64°33′779″ S–45°27′440” to 63°16′554”: Antarctic Peninsula and South Shetland Islands area	*E. coli*, *S. enterica*, *Enterobacter aerogenes*, *Citrobacter freundii*, *S. marcescens*, *Shigella* sp., *S. aureus*, *Staphylococcus epidermidis*, *B. subtilis*, *Xanthomonas* sp., *Erwinia* sp.	[63]
*Lysobacter oligotrophicus*	Bottom of freshwater lake	Skarvsnes region	*E. coli*, *Lysobacter enzymogenes*, *Rhodoligotrophos appendicifer*, *Saccharomyces cerevisiae*.	[64]
*Methylobacterium* sp.	Soil	62°24′ S, 59°47′ W: Barrientos Island	*S. aureus*, *C. albicans*	[57]
*Paracoccus* sp.	Soil	62°24′ S, 59°47′ W: Barrientos Island	*C. albicans*	[57]
*Pseudoalteromonas* sp.	Sea water	72°19′ S to 74°53′ S–163°48′ E to 70°16′ E: Stations Mergellina Santa Maria Novella, Tiburtina, Road Bay, Gerlache Inlet, Evans Cove, Inexpressible Island, Cape Hallet, and Tethys Bay	*E. coli*, *M. luteus*, *P. mirabilis.*	[14]
			*B. cepacia* complex pathogens	[65]
*Pseudoalteromonas haloplanktis*	Sea water	66°40′ S; 140° 01′ E: French Antarctic station Dumont d’ Urville, Terre Adélie	Biofilms formation of *S. epidermidis*	[66]
			*B. cepacia* complex pathogens	[67,68]
*Pseudomonas* sp.	Soil	72°19′ S to 77°83′ S, 16° 55′ E to 17° 16′ E: Cape Hallett, Edmonson Point, Kay Island, Cape Russell, Lake Hoare, Harrow Peaks, Crater Circe, Battleship Promontory, Mount, McGee, Mount Rittmann, Mount Melbourne	*Listeria* spp., *B. thermosphacta*	[17]
	Sub-sea sediment	74°41′36.96′ S, 164°6′42.12′ E: Terranova Bay, Ross sea	*B. cepacia* complex pathogens	[54]
	Marine sediment	62°58′788” to 62°05′948″ S–60°33′464” to 58°23′622″ W: Deception Island, Martel Bay, King George Island and Punta Hannah sediment	*M. luteus*, *S. aureus*	[38]
	Benthic microbial mat	Larsemann Hills, Vestfold Hills and McMurdo Dry Valleys	*S. aureus*, *E. faecium*, *E. coli*	[55]
	Ornithogenic soil	62°59′ S, 60°34′ W: Whalers Bay on Deception Island, South Shetland Islands and 65°14′44.6″ S, 64°15′26″ W: Galindez Island, Argentine Islands	*B. subtilis*, *B. cereus*, *Sarcina lutea*, *M. luteus P. aeruginosa*, *E. coli*, *A. johnsonii*, *X. oryzae*, *Candida* sp., *Cryptococcus* sp.	[56]
	Soil	62°09′30.0″ S, 58°56′15.2″ W: King George Island	*E. coli*, *S. enterica serovar Typhimurium*, *K. pneumoniae*, *E. cloacae*, *B. cereus*, *V. parahaemolyticus*	[69]
*Pseudomonas fragi*	Water column, rock surfaces	62°12′ S, 58°57′ W: King George Island	*Flavobacterium psychrophilum* biofilm	[70]
*Psychrobacter*	Sub-sea sediment	74°41′36.96″ S, 164°6′42.12″ E: Terranova Bay, Ross sea	*B. cepacia* complex pathogens	[54]
	Benthic microbial mat	Larsemann Hills, Vestfold Hills and McMurdo Dry Valleys	*S. aureus*, *E. coli*	[55]
*Shewanella* sp.	Lake ponds benthic microbial mats	Larsemann Hills, Vestfold Hills andMcMurdo Dry Valleys	*S. aureus*, *E. coli*	[55]
*Sphingomonas* sp.	Soil	62°24′ S, 59°47′ W: Barrientos Island	*C. albicans*	[57]
**Cyanobacteria**				
*Leptolyngbya antarctica*	Benthic microbial mat	Larsemann Hills, Bølingen Islands, Vestfold Hills, Rauer Islands, and the McMurdo Dry Valleys	*S. aureus*	[71]
*Nostoc* sp.	Benthic microbial mat	Larsemann Hills, Bølingen Islands, Vestfold Hills, Rauer Islands, and the McMurdo Dry Valleys	*M. tuberculosis*, *S. aureus*, *S. typhi*, *P. aeruginosa*, *E. aerogenes*, multidrug-resistant strains of *E. coli*, *C. neoformans*	[71,72]
*Phormidium priestleyi*	Benthic microbial mat	Larsemann Hills, Bølingen Islands, Vestfold Hills, Rauer Islands, and the McMurdo Dry Valleys	*S. aureus*, *A. fumigatus*, *Cryptococcus neoformans*	[71]
*Phormidium murrayi*	Benthic microbial mat	Larsemann Hills, Bølingen Islands, Vestfold Hills, Rauer Islands, and the McMurdo Dry Valleys	*S. aureus*	[71]
*Pseudophormidium* sp.	Benthic microbial mat	Larsemann Hills, Bølingen Islands, Vestfold Hills, Rauer Islands, and the McMurdo Dry Valleys	*S. aureus*, *C. neoformans*	[71]
**Firmicutes**				
*Bacillus* sp.	Sea water	50°76′ W, 61°16′ S	*Paecilomyces variotii*, *Colletotrichum gloeosporioides*, *Fusarium oxysporum*, *Trichoderma viride*, *Rhizoctonia solani*	[73]
	Ornithogenic soil	62°59′ S, 60°34′ W: Whalers Bay on Deception Island, South Shetland Islands and 65°14′44.6′ S, 64°15′26″ W: Galindez Island, Argentine Islands	*B. subtilis*, *B. cereus*, *P. aeruginosa*, *E. coli*, *A. johnsonii*, *Candida* sp.	[56]
	Soil	62°04′ S, 58°21′ W: King George Island	Methicillin resistant *S. aureus*, *C. albicans*	[74]
*Enterococcus* sp.	Soil	69°21.68′ S, 76°07.76′ E; 69°21.68′ S, 76°07.70′ E and 69º22.433′ S, 76°08.940′ E: Penguin rookeries Larsemann Hills, East Antarctica	Multidrug-resistant strains of *C. albicans*	[75]
*Planococcus* sp.	Soil	72°19′ S to 77°83′ S–16° 55′ E to 17° 16′ E: Cape Hallett, Edmonson Point, Kay Island, Cape Russell, Lake Hoare, Harrow Peaks, Crater Circe, Battleship Promontory, Mount, McGee, Mount Rittmann, Mount Melbourne	*Listeria* spp., *B. thermosphacta*	[17]
*Sporosarcina* sp.	Ornithogenic soil	62°59′ S, 60°34′ W: Whalers Bay on Deception Island, South Shetland Islands and 65°14′44.6″ S, 64°15′26″ W: Galindez Island, Argentine Islands	*B. subtilis*, *B. cereus*, *S. lutea*, *P. aeruginosa*, *E. coli*, *A. johnsonii*, *X. oryzae*, *Candida* sp., *Cryptococcus* sp.	[56]
		62°04′ S, 58°21′ W: King George Island	Methicillin resistant *S. aureus*, *C. albicans*	[74]
**Bacteroidetes**				
*Flavobacterium* sp.	Freshwater	70°45′52.3″ S, 11°37′10.7″ E: Lake Podprudnoye, Schirmacher Oasis, Dronning Maud Land	*M. smegmatis*, *M. tuberculosis*	[62]
*Pedobacter* sp.	Soil	62°09′30.0″ S, 58°56′15.2″ W: King George Island	*E. coli*, *S. enterica serovar Typhimurium*, *S. enterica serovar Typhi*, *K. pneumoniae*, *E. cloacae*, *B. cereus*	[69]
